# Hybrid Nanocomposite Solid Electrolytes (n-C_4_H_9_)_4_NBF_4_–MgO

**DOI:** 10.3390/ijms241310949

**Published:** 2023-06-30

**Authors:** Yulia Mateyshina, Ivan Stebnitskii, Danil Shivtsov, Ekaterina Ilyina, Artem Ulihin, Andrey Bukhtiyarov, Nikolai Uvarov

**Affiliations:** 1 Institute of Solid State Chemistry and Mechanochemistry SB RAS, Kutateladze St. 18, Novosibirsk 630090, Russia; yuliam@solid.nsc.ru (Y.M.); i.stebnitskii@g.nsu.ru (I.S.); ulikhin@solid.nsc.ru (A.U.); 2 Department of Natural Sciences, Novosibirsk State University, Pirogova St.1, Novosibirsk 630090, Russia; 3 Boreskov Institute of Catalysis SB RAS, Lavrentiev Ave. 5, Novosibirsk 630090, Russia; danil@catalysis.ru (D.S.); evi@catalysis.ru (E.I.); avb@catalysis.ru (A.B.)

**Keywords:** tetrabutylammonium tetrafluoroborate, magnesium oxide, hybrid nanocomposites, amorphous interface-stabilized phase, amorphous layer thickness, ionic conductivity

## Abstract

Hybrid nanocomposite materials Bu_4_NBF_4_–MgO were obtained using a nanocrystalline MgO with a specific surface area of 324 m^2^/g and the grains size of 5.1 nm. As a result of the strong adhesion, the salt transforms into an interface-stabilized amorphous state within the thin layer near the interface. The analysis of the DSC data allowed one to estimate the concentration and the thickness of this amorphous layer as 4.8 nm. The amorphous interface phase has an enhanced ionic conductivity. As a result, conductivity of the nanocomposite increases with the concentration of the amorphous phase and reaches 1.1 × 10^−3^ S/cm at 150 °C at a concentration of the MgO additive x = 0.90 corresponding to the maximum content of the amorphous phase. The conductivity of the nanocomposite is by three orders of magnitude higher than the conductivity of pure Bu_4_NBF_4_. The nanocomposites are electrochemically stable up to 2.5 V. At high concentrations of MgO when the total volume of the salt is small the composites become nano- and mesoporous.

## 1. Introduction

Hybrid materials are a new promising class of materials which have many applications in many areas such as construction materials, energy, electronics, optics, ionics, environment, and biomedicine, et al. [1,2,3,4,5,6,7]. Among the hybrid materials, polymer nanocomposites obtained as a result of the combination of a polymeric matrix and an inorganic nanomaterial are of special interest as they have improved mechanical strength, toughness and stiffness, thermal conductivity, superior flame retardancy, and a higher barrier to moisture and gases [8]. Organic salts are possible alternatives to polymer systems. They are characterized by a great variety of possible crystal structures, polymorphs, and may contain different cations or anions with simple or more complicated structures. A remarkable feature of organic salts is an easy reorientation of organic groups, especially in high-temperature phases [9,10,11]. Organic salts are ionic liquids in a molten state. A relatively high ionic conductivity (above 10^−6^ S/cm) has been observed in high-temperature phases of many organic ionic compounds, such as substituted ammonium salts with various cations and anions [9,10,11,12,13,14,15,16,17,18,19,20], substituted piperidinium [21,22], pyrrolidinium [9,23,24], and imidazolium [9,10,11,25] salts. Quaternary ammonium salts comprise a class of relatively stable compounds that may be regarded as a suitable model system for the investigation of correlations between structure, thermodynamic properties, molecular disordering, and ionic conductivity. Among quaternary ammonium compounds, tetra-n-butylammonium (Bu_4_N^+^) salts are of special interest as they are typical ionic liquids in the molten state and have high-temperature polymorphs which may be attributed to plastic phases. The relatively high ionic conductivity was observed in high-temperature phases of Bu_4_N^+^ salts (n-C_4_H_9_)_4_NI [19], (n-C_4_H_9_)_4_NBF_4_ [26], (n-C_4_H_9_)_4_NBr [27].

In all cases, ionic conductivity is observed in high-temperature phases, whereas low-temperature phases are insulators. In order to elevate the ionic conductivity of organic salts two possible approaches may be used:

(i) classical method of doping the salt with heterovalent dopants. Unfortunately, there are problems with synthesis of organic compounds with doubly charged cations or anions of appreciable ionic size which might form solid solutions;
(ii) heterogeneous doping technique, well-known for inorganic composites, nanocomposites, and heterostructures [28,29,30]. In this case, the addition of the nanocrystalline or nanoporous additive to the ionic salt results in a strong enhancement of the ionic conductivity of the salt.

The second approach is general and is applicable when the adhesion energy of the salt to the surface of the additive is sufficiently high. In this case, the ionic salt may spread on the surface of the additive. If the additive is nanocrystalline or nanoporous, then a nanocomposite may form simultaneously as a result of the spreading. In contrast to inorganic nanocomposites, there are only a few data on hybrid organic–inorganic nanocomposite solid electrolytes. Nevertheless, recently a relatively high ionic conductivity was observed in Bu_4_N^+^ salts with the addition of metal-organic framework [31], nanocrystalline alumina [32], nanodiamonds [33], or nanoporous silica [34] It was found that the heterogeneous doping leads to the formation of hybrid nanocomposites. However, the influence of the morphology and surface chemical activity of the additives on the transport and thermodynamical properties of the organic salt in nanocomposites requires systematic study. In the present work, a hybrid nanocomposite solid electrolyte based on tetra-n-butylammonium tetrafluoroborate, (n-C_4_H_9_)_4_BF_4_ (Bu_4_NBF_4_) with the additives of nanocrystalline magnesium oxide Bu_4_NBF_4_–MgO were prepared and their thermodynamic, structural, and transport properties investigated in detail. Magnesium oxide has simultaneously a high surface area and high surface basicity. Therefore, it would be of interest to compare the properties of the Bu_4_NBF_4_–MgO with ones obtained using other additives.

## 2. Results and Discussion

### 2.1. X-ray Diffraction, Electron Microscopy and XPS Studies

**Figure 1:**
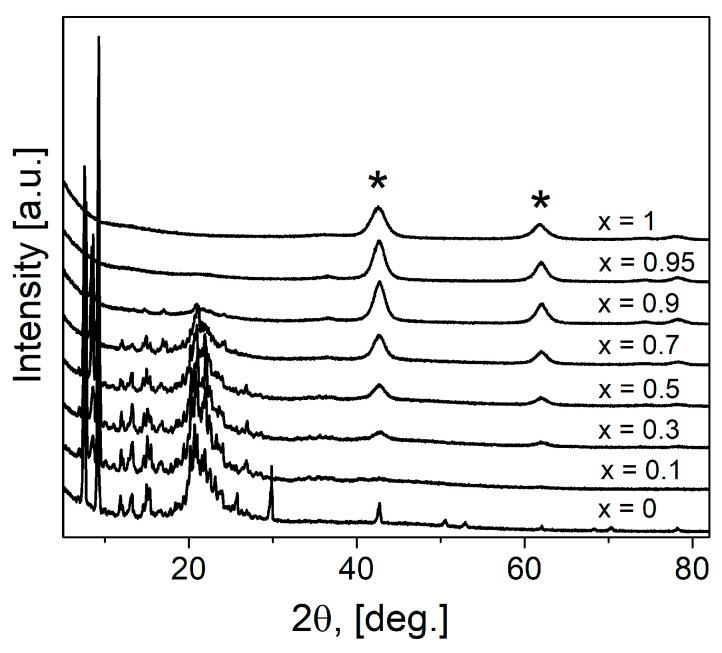
X-ray diffraction patterns for pure components and (1-x)Bu_4_NBF_4_–xMgO composites. The symbol “*” marks reflections of MgO.

All obtained (1-x)Bu_4_NBF_4_–xMgO composites (where x is the molar fraction) were studied by the X-ray diffraction analysis over the 15° < 2θ < 90° angle range (Figure 1). As seen, the reflections belonging to the organic and inorganic phases are strongly separated. In the X-ray diffraction patterns, the reflections relating to MgO (space group *Fm*3*m*, PDF 45-946) are marked by the “*” symbol; they are observed for compositions with magnesium oxide concentration x > 0.3. All reflections attributed to MgO are broadened. The grains size (or more correctly, the coherent domains size) of magnesium oxide *L* as estimated from the Scherrer equation,

(1) $$L=\frac{K\lambda }{\beta cos\left(\theta \right)}$$

where *λ* is the X-ray wavelength; *β* is the line broadening at the half the maximum intensity; *θ* is the Bragg angle; *K* is a parameter close to unity. The grain size value estimated from X-ray data is equal to 4.7 ± 0.4 nm. This value may be estimated independently from the specific surface area value (assuming that the sample consists of identical cubic particles) using a relation:

(2) $$L=\frac{6}{\rho \cdot S_{sp}}$$

where *ρ* = 3.58 g/cm^3^ is the MgO density and *S_sp_* = 324 m^2^/g is the specific surface area of MgO. The value of *L* estimated from Equation (2) is equal to 5.1 nm and not strongly differs from one obtained from X-ray data. Thus, the magnesium oxide taken as an additive in the present work is indeed nanocrystalline.

The reflections of tetrabutylammonium tetrafluoroborate correspond to the low-temperature phase [35]. Several reflections disappear in composites after heating, the same picture was observed in pure salt after melting and cooling to room temperature. As reported earlier [35] the structure of the low-temperature phase Bu_4_NBF_4_ belongs to a monoclinic space group of P2_1_/c. The reflections attributed to Bu_4_NBF_4_ can be seen on the diffraction pattern up to the composition with x = 0.7. No marked broadening of the reflections relating to the salt phase was observed.

**Figure 2:**
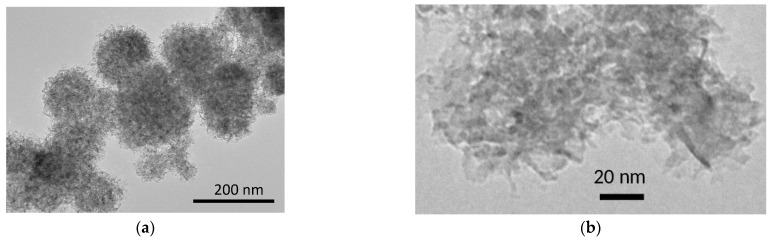
High-resolution electron microscopy image of nanocrystalline MgO at low (**a**) and high (**b**) magnification.

High-resolution electron microscopy image of nanocrystalline MgO used as an additive in the composites under study is shown in Figure 2. It is seen that the sample consists of nanoparticles with a broad size distribution in the range 3–10 nm that agrees with the values estimated from X-ray diffraction peak broadening and the specific surface area. The nanoparticles form a large spherical aggregate 100–200 nm in size.

Samples Bu_4_NBF_4_, MgO, and the composite 0.08Bu_4_NBF_4_-0.92MgO were studied by XPS spectroscopy. In the spectra of Bu_4_NBF_4_ only lines related to boron, carbon, nitrogen, and fluorine were observed, the MgO spectra included only magnesium and oxygen lines, whereas in the spectra of the composite, all the lines mentioned were presented. No lines of other elements were found within the sensitivity of the XPS method. The regions C1s, N1s, O1s, Mg2p, and F1s were measured to determine the chemical (charge) state and the ratio of atomic concentrations of elements on the surface of the samples. The values of the binding energies of all peaks were calibrated by the position of the C1s photoelectron line (284.7 eV), which is characteristic of C–C/C–H bonds [36].

**Figure 3:**
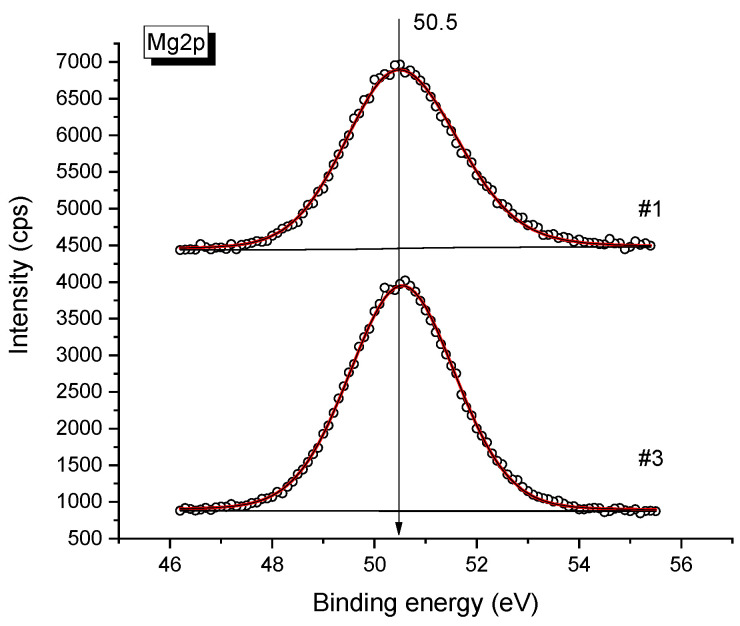
XPS spectra of Mg2p obtained for pure MgO (upper spectra) and 0.08Bu_4_NBF_4_-0.92MgO composite (bottom spectra). Points are experimental data, lines are fitting curves.

From the analysis of the shape and half-width of the Mg2p spectrum measured for pure MgO and the magnesium oxide in the 0.08Bu_4_NBF_4_-0.92MgO samples (Figure 3), it may be concluded that in both samples the binding energy of the Mg2p line of about 50.5 eV, which is characteristic of Mg^2+^ in the MgO oxide [36,37].

The shape and half-width of the line also remain unchanged, which indicates that the state of Mg does not change upon mixing. Also, the O/Mg atomic ratio is one for both samples (see Table 1 below), this value corresponds to the MgO stoichiometry.

From XPS data the relative content of elements on the surface of samples were estimated in the form of atomic concentrations (Table 1). The integrated line intensities were measured from the areas of the respective regions (C1s, N1s, O1s, Mg2p, and F1s). The determination of the relative content of elements on the surface of the samples and the ratio of their atomic concentrations was carried out by the integral intensities of photoelectron lines corrected for the corresponding atomic sensitivity coefficients [38].

### 2.2. Thermal and Adsorption Properties

**Figure 4:**
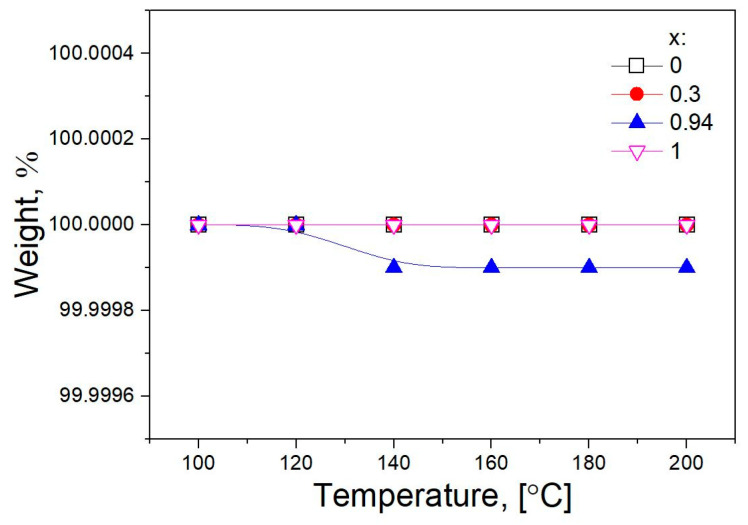
Dependences of weight loss of pure components and (1-x)Bu_4_NBF_4_–xMgO composites (x = 0.3 and 0.94) on temperature.

Before experiments, the study of the weight loss of the composite was carried out in real time using a McBain balance. The samples were heated in an argon flow at temperatures of 100, 120, 140, 160, 180, and 200 °C for 10 min at each temperature. The total flow rate of argon is 10 l/h. The change in the mass of the sample was monitored using a cathetometer. At the end of the experiment, the reactor was cooled to room temperature in an argon flow. As seen from Figure 4, heating the samples up to 200 °C does not accompany by a weight loss within the 0.01 weight percent. Therefore, the samples under study are stable in conditions of further experiments.

**Figure 5:**
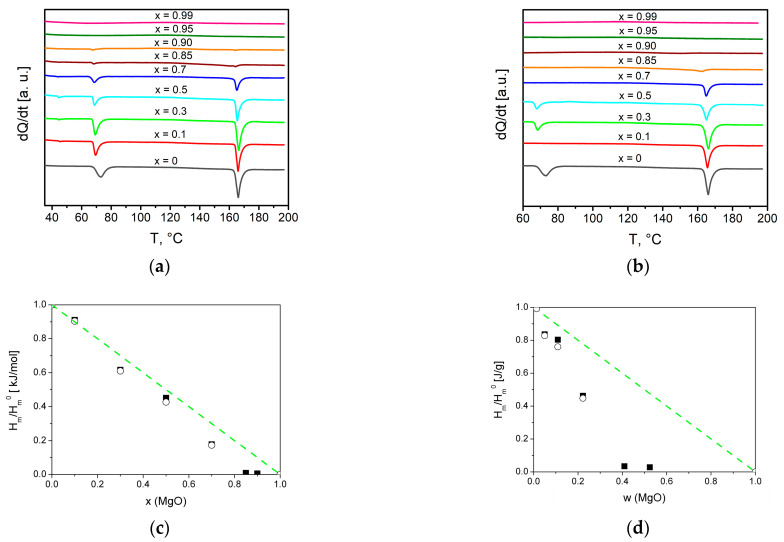
DSC curves for composites (1-x)Bu_4_NBF_4_–xMgO obtained on the first (**a**) and second (**b**) heating. Experimental values of the relative change in the heat effect due to melting of tetrabutylammonium tetrafluoroborate (squares—1 heating, circles—2 heating) in comparison with the dependences expected for random mixture of the components (lines) for molar (**c**) and weight (**d**) fraction of MgO.

The composites formation process was investigated in situ by the DSC method, the results are presented in Figure 5. On the first heating of initial mixtures (1-x)Bu_4_NBF_4_–xMgO, two endothermic peaks were observed corresponding to phase transitions from the low- to the high-temperature phase of Bu_4_NBF_4_ at 60–70 °C and melting of the ionic salt at ~160 °C.

On the second heating thermal effects of both the polymorphous transition and the melting of the ionic salt in the composites strongly decreased.

A similar effect observed earlier in many systems of the ‘ionic salt–oxide’ type is caused by two phenomena: spreading of the ionic salt on the oxide surface and amorphization of the salt in the vicinity of the salt/oxide interface [28,29,30]. The absence of the thermal effects in the composites with x > 0.85 (corresponding to the weight fraction *w* = 0.40) on the second heating can be caused by a complete transformation of Bu_4_NBF_4_ to an interface-stabilized amorphous state. In parallel to the decrease in the phase transition enthalpies, a shift in the phase transition temperatures takes place. Such a shift is weak for melting and much stronger for the polymorphous phase transition. This phenomenon may be explained by the size effect that appears when the effective grain size of the salt becomes small as a result of the spreading of the salt along the oxide surface [28,29,30]. It is surprising that the effect is observed on the first heating. Possibly, the spreading of Bu4NBF4 on the magnesium oxide surface takes place even at room temperature during the preparation of the initial mixtures.

The deviation of experimental data from linear dependences H_m_/H_m_^0^ [kJ/mole] = 1 − *x* and H_m_/H_m_^0^ [J/g] = 1 − *w* may be explained by the presence of an interface-stabilized amorphous phase which does not give diffraction pattern (as seen from Figure 1) and has diffused melting point. The dependences of the normalized melting enthalpy on the concentration of the MgO additive may be qualitatively described in terms of the model proposed earlier [28,29,30]. Assuming that the volume fraction of the MgO in the composite is equal to *f* and an amorphous layer of the salt with the thickness of *λ* exists on the surface of MgO, the volume fraction of the amorphous interface *f_S_* phase for random distribution of the components may be estimated as:

(3) $$f_{S}=\beta \frac{\lambda }{L}\cdot f\cdot \left(1-f\right)\;(f_{S}\leq 1\;-\;f),$$

where *L* is the grains size of MgO; *β* is the geometric parameter. The volume fraction *f_S_* can be converted to the weight *w_S_* or molar *x_S_* fractions of the interface phases using the relations:

(4) $$w_{S}=\beta \frac{\lambda }{L}\cdot \frac{f_{S}}{f+\left(1-f\right)\cdot \frac{\rho_{1}}{\rho_{2}}}\;(w_{S}\leq 1\;-\;w),$$

(5) $$x_{S}=\beta \frac{\lambda }{L}\cdot \frac{f_{S}}{f\cdot \frac{M_{2}\rho_{1}}{M_{1}\rho_{2}}+\left(1-f\right)}\;(x_{S}\leq 1\;-\;x),$$

where *ρ*_1_ = 1.0 g/cm^3^ and *ρ*_2_ = 3.58 g/cm^3^ are densities of Bu_4_NBF_4_ and MgO, respectively; *M*_1_ = 329.3 g/mol and *M*_2_ = 40.3 g/mol are their molecular weights. Values of MgO volume fraction may be expressed via weight or molar fraction values as:

(6) $$f=\frac{w}{w+\left(1-w\right)\cdot \frac{\rho_{2}}{\rho_{1}}}=\frac{x}{x+\left(1-x\right)\cdot \frac{M_{1}\rho_{2}}{M_{2}\rho_{1}}}.$$

The fraction of the bulk crystalline phase in the composite is equal to

(7) $$f_{bulk}=1-f-f_{S}\;(f_{bulk}\;>\;0),$$

(8) $$w_{bulk}=1-w-w_{S}\;(w_{bulk}\;>\;0),$$

(9) $$x_{bulk}=1-x-x_{S}\;(x_{bulk}\;>\;0),$$

At high concentration of the MgO additive practically all the ionic salt becomes amorphous. In this case *f_bulk_* (as well as *w_bulk_* and *x_bulk_*) is equal to zero and *f_S_* = 1 − *f* (as well as *w_S_* = 1 − *w* and *x_S_* = 1 − *x*).

**Figure 6:**
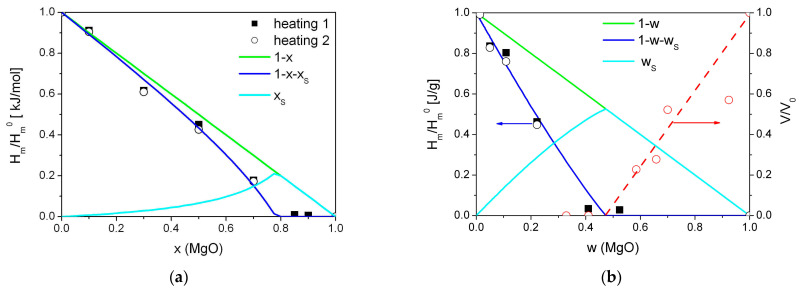
Experimental values of the relative change in the heat effect due to melting of tetrabutylammonium tetrafluoroborate in (1-x)Bu_4_NBF_4_–xMgO composites (squares—1 heating, circles—2 heating) in comparison with the dependences expected for random mixture of the components (lines) for molar (**a**) and weight (**b**) fraction of MgO. Red symbols are experimental values of the normalized pore volume obtained from adsorption data, red line is a guide for eye.

Relative values of the heat of melting (normalized to the melting enthalpy of pure Bu_4_NBF_4_) were fitted to the theoretical curves obtained using Equations (3)–(9). As seen, the fitting curves fairly describe experimental data in both scales, molar, and weight fraction, as shown in Figure 6a,b, respectively.

The best fit was obtained for the parameters *β*(*λ*/*L*) = 5.0. Assuming that for a randomly mixed composite the geometric parameter *β* is equal to 6 and the grain size of MgO is nearly 4.8 nm, the thickness of the amorphous interface layer is nearly 4 nm.

**Figure 7:**
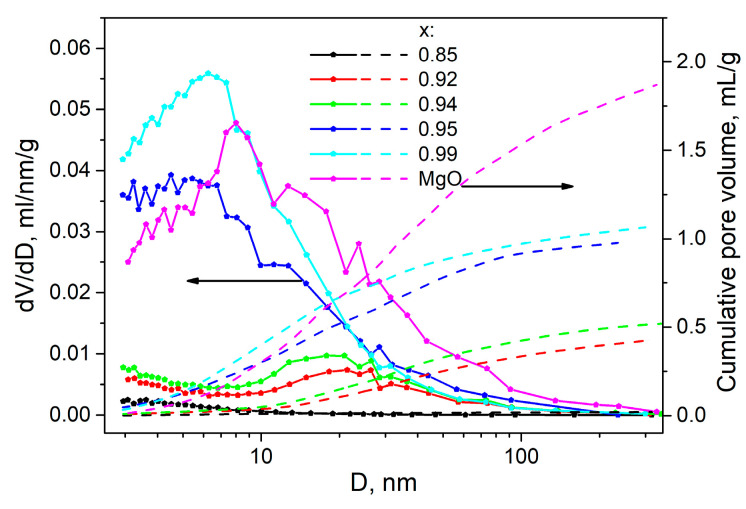
Experimental values of the pore distribution and the total cumulative pore volume in pure MgO and (1-x)Bu_4_NBF_4_–xMgO composites as a function of the pore size.

At high MgO concentrations, the salt Bu_4_NBF_4_ occurs in the amorphous state. There is a question, how does the composite morphology change with further increase in the oxide content? In order to answer this question, nitrogen adsorption experiments were carried out. Figure 7 shows the pore distribution and the total number of pores as a function of the pore diameter in the (1-x)Bu_4_NBF_4_–xMgO composites. One can see that pure MgO has a broad pore distribution with the mean pore size of 8 nm. The addition of Bu_4_NBF_4_ results in the following effects:

- at low salt content (0.95 < x < 1), the mean pore size decreases that may be explained by filling small interparticle pores of MgO by the salt. The filling proceeds via spreading the salt along the pore surfaces;
- at higher loading of the salt (0.85 < x < 0.94) all small pores (with the size less than 10 nm) are completely filled and only large pores (nearly 20 nm in size) remain unfilled.
- at higher concentration of the salt (x < 0.85), no pores are found by the adsorption measurements;

The increase in the concentration of the salt leads to a monotonical decrease in the total cumulative pore volume from the value of 1.87 cm^3^/g for pure MgO to zero for the sample with x = 0.85. It allows one to plot the dependence of relative porosity (normalized to the total pore volume of MgO) on the concentration in the composites under study. Such dependence is represented in Figure 6b as a function of the weight fraction of the oxide.

One can see that the increase in the MgO content is accompanied by filling all the pores of the oxide additive and the amorphization of the salt. When the concentration of the additive exceeds some limiting concentration, the total volume of the salt becomes comparable or less that the pore volume of the additive. It leads to the increase in porosity of the samples. Similar phenomena were observed nanocomposites based on mesoporous matrices [31,39].

### 2.3. Ionic Conductivity and Electrochemical Stability

**Figure 8:**
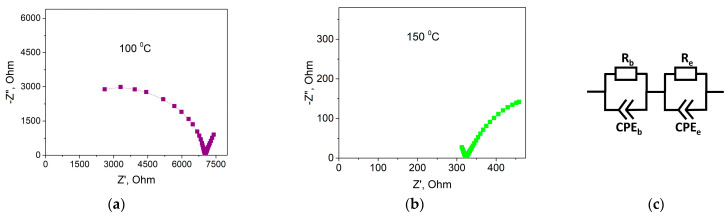
Plots of the complex impedance at 100 °C (**a**) and 150 °C (**b**) for the 0.1Bu_4_NBF_4_–0.9MgO composite. Equivalent electrical circuit used for the data analysis, where *R*_b_ is the bulk resistance, *R*_e_ is the charge transfer resistance, and CPE_b_ and CPE_e_ are the constant phase elements (**c**).

Conductivity of composite solid electrolytes (1-x)Bu_4_NBF_4_–xMgO (0 ≤ x < 1) were investigated using the electrochemical impedance spectroscopy technique. Figure 8 shows typical Nyquist plots obtained for 0.1Bu_4_NBF_4_–0.9MgO composite at 100 and 150 °C.

In the impedance plots, there are two oblate semicircles corresponding to the bulk impedance at high frequencies and the electrode impedance at low frequencies. To calculate the impedance parameters, we chose optimal equivalent electrical circuit presented in Figure 8c. The circuit comprised the bulk impedance including the active resistance *R_b_* and the constant phase element CPE_b_ and the electrode impedance including the charge transfer resistance *R*_e_ and the constant phase element CPE_e_. From the analysis of the Nyquist plots, the values of the resistance *R_b_* and the conductivity σ = *R_b_*^−1^*·d·S*^−1^ (where *d* is the pellet thickness; *S* is the electrode surface area) were calculated for each temperature and the temperature dependences of conductivity were plotted.

**Figure 9:**
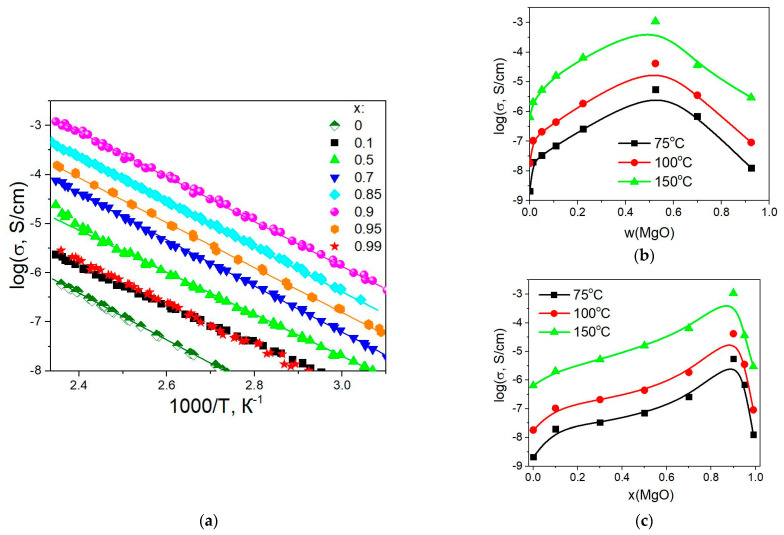
Temperature dependences of electrical conductivity for composites (1-x)Bu_4_NBF_4_–xMgO (**a**) and the variation of conductivity with the weight (**b**) and molar (**c**) fraction of MgO at the temperatures of 75, 100, and 150 °C.

Temperature dependences of conductivity for the composites (1-x)Bu_4_NBF_4_–xMgO (0 ≤ x < 1) are presented in Figure 9a. The conductivity monotonically increases with the temperature and is well reproducible in the heating–cooling cycles. Conductivity values obey the Arrhenius law σ = (*A*/*T*)·exp (*E*_a_/*RT*), where *A* is the pre-exponential factor and *E*_a_ is the activation energy. The calculated conductivity parameters *E*_a_ and log(*A*) are shown in Table 2. Figure 9b,c shows the dependence of the conductivity of the composites on weight and molar fractions of MgO at temperatures of 75, 100, and 150 °C.

It is seen that the conductivity of the composites increases monotonically with the concentration of the inert oxide additive up to x = 0.9 (corresponding to the weight fraction of *w* = 0.52 and the volume fraction of *f* = 0.24). Maximum conductivity σ = 1.1 × 10^–3^ S/cm at 150 °C observed for the composite 0.1Bu_4_NBF_4_–0.9MgO. The conductivity of the composite is by three orders of magnitude higher than the conductivity of pure Bu_4_NBF_4_. Further increase in heterogeneous additive concentration leads to a decrease in conductivity. Typically, the maximum electrical conductivity values for composite solid electrolytes are observed in the range concentration of oxide inert additive ~30–60 vol.%, at which the contact area between components of the composite reaches its maximum value. This value of the volume fraction of the inert additive is typical of the composite solid electrolytes [28,29,30], the conductivity of which is due to the presence of the ionic salt/oxide interface. Probably, in the case of composites with magnesium oxide as an inert heterogeneous additive, it is also necessary to take into account the effect of oxide porosity on the conductivity of composites.

**Figure 10:**
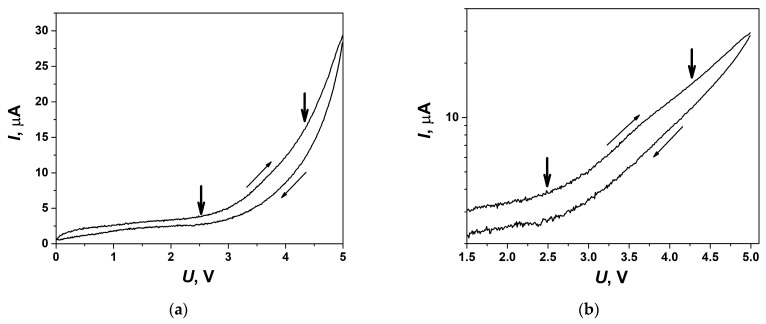
CVA curve obtained for electrochemical cell Ni/0.1Bu_4_NBF_4_–0.9MgO/Ni at 140 °C on the second cycle at the voltage sweep rate of 5 mV/s and represented in a linear scale (**a**) and as a Tafel plot (**b**). Bold arrows point the approximate values of voltage corresponding to the electrochemical processes.

In order to test the electrochemical stability of the composite solid electrolytes under study, volt-ampere dependencies were analyzed. The experiments were carried out in the symmetrical cell with Ni electrodes in a fore-vacuum at a temperature of 140 °C in the voltage range of 0–5 V. Cyclic volt-ampere (CVA) curve obtained for the cell at the scan rate of 5 mV/s is shown in Figure 10a. A noticeable increase in the current observed at the voltage above nearly 2.5 V is due to the electrochemical process taking place at the electrodes. This value might be interpreted as the electrochemical decomposition voltage of the salt in the 0.1Bu_4_NBF_4_–0.9MgO composite. However, a comparison with the CVA data obtained by us earlier for Bu_4_NBF_4_–Al_2_O_3_ composites [32] shows that the decomposition voltage of the Bu_4_NBF_4_ salt exceeds 4 V, i.e., essentially higher than the value of 2.5 V estimated from CVA curve for 0.1Bu_4_NBF_4_–0.9MgO composite.

In order to understand the reason for such discrepancy, the CVA curve was represented in Tafel coordinates log(*I*) vs. *U*, such a plot is shown in Figure 10b. From this plot, it is clearly seen that two electrochemical processes take place in the cell: the first process at the voltage of 2.5 V, whereas the second one starts at the voltage range of 4–4.5 V. When analyzing the data in a linear scale it is difficult to observe the second process and determine its characteristic voltage. The Tafel plot allows one, at least roughly, to separate both processes. The electrochemical process taking place at 2.5 V may be explained by the electrolysis of OH groups on the MgO surface. Though typical voltage values for such processes should be below 2 V, this value can be shifted due to the electrode polarization. Another reason of this process is the electrochemical decomposition of BF_4−x_OH_x_^−^ anions which may be formed as a result of partial hydrolysis of BF_4_^−^ anions in the vicinity of partially hydrated MgO surface. The concentration of the OH groups on the surface of MgO seems to be higher than on Al_2_O_3_ surfaces leading to much stronger influence of the electrochemical process at 2.5 V in Bu_4_NBF_4_–MgO compared to Bu_4_NBF_4_–Al_2_O_3_ ones provided that the decomposition voltage of the Bu_4_NBF_4_ salt remains equal to nearly 4–4.5 V in both the composites.

## 3. Materials and Methods

Ionic salt (C_4_H_9_)_4_NBF_4_ (hereafter, Bu_4_NBF_4_) was recrystallized and dried at 120 °C. Magnesium oxide (space group Fm3m, PDF 45-946, specific surface area 324 m^2^/g, manufactured by Boreskov Institute of Catalysis, Novosibirsk, Russia) was preliminarily conditioned at 500 °C for 2 h for dehydration. Composites (1-x)Bu_4_NBF_4_–xMgO were prepared from pre-dehydrated components in wide-range compositions (0 < x < 1) with the concentration step (x) of 0.05–0.5 M fraction. The starting components were mixed in an agate mortar. The resulting mechanical mixture was heated in an oven at 160 °C for 30 min, the procedure was repeated 3 times to evenly distribute the components in the composite. The phase composition of the solid composite electrolytes was studied by the X-ray diffraction analysis by using a D8 Advance diffractometer (Bruker, Mannheim, Germany) with a Lynx-Eye one-dimensional detector and Kβ-filter, with the use of CuKα-radiation, over the 15 < 2θ° < 90° angle range in increments of Δ2θ = 0.0195°. High-resolution electron microscopy studies were carried out on a Hitachi HT7700 TEM equipped with an EDX Bruker X-flash 6T/60 spectrometer at an amplified voltage of 100 kV. The powdered samples were deposited as a suspension in ethanol on TEM grids, dipped in perforated carbon film. The XPS measurements were carried out on a SPECS photoelectron spectrometer (SPECSGROUP, Berlin, Germany) using Al Kα radiation (hν = 1486.6 eV, 150 W). The binding energy scale (E_b_) was preliminarily calibrated by the position of the peaks of the core levels of gold and copper: Au 4f7/2 (84.0 eV) and Cu 2p3/2 (932.67 eV). The pressure of residual gases during measurements did not exceed 8 × 10^−9^ mbar. The samples were applied to a standard holder on double-sided conductive copper adhesive tape Scotch 3M© (Shenzhen Yousan Technology Co., Shenzhen, China). The obtained spectral information was processed using the XPSPeak 4.1 program. The thermal properties of the composites were studied on DSK-500 scanning calorimeter (SamSTU, Samara, Russia). The weight loss of the composites was measured using a flow gravimetric setup equipped with a McBain balance with an accuracy of 0.01%. A weighed sample of 100 ± 50 mg was placed in a foamed quartz basket, suspended by a quartz spring, and loaded into a flow-through quartz reactor. The pore structure was studied by nitrogen adsorption–desorption at 77 K. Isotherms were measured using an automatic adsorption analyzer Sync 200 (3P INSTRUMENTS, Odelzhausen, Germany). Before measurements, all samples were degassed in a vacuum of less than 1 Pa at 200 °C for 3 h. All calculations were performed using the 3P Sync software version 10.03.08.00. The pore volume was calculated at a final relative pressure of 0.995. A branch of the adsorption isotherm was used to calculate the pore size distribution. The conductivity was measured at pellets with a diameter of d = 6.2–6.3 mm and thickness of h = 0.7–1.5 mm, compacted at a pressure of 15 ± 5 MPa with silver powder electrodes. The electrical measurements were carried out in a fore-vacuum (5 × 10^−2^ Torr) over a temperature range of 25–150 °C in the stepwise isotherm mode, by using a two-electrode circuit. An LCR Hewlett Packard HP 4284A precision meter was used over 20 Hz–1 MHz ac frequency range with a signal amplitude of 10 mV. The conductivity values were calculated for each temperature by analyzing Nyquist impedance plots −Z″ = f(Z′). The results were processed using EIS Spectrum Analyzer software (Belarus) [40]. The total ionic conductivity was calculated as σ = d/(R⋅S), where d is the sample thickness and S is the electrode area. Volt-ampere curves were recorded using a Zive SP2 Electrochemical Workstation (Zive, New York, NY, USA) in the symmetrical cell with nickel electrodes closely attached to the solid electrolyte pellet in a fore-vacuum.

## 4. Conclusions

In this work, hybrid nanocomposite materials Bu_4_NBF_4_−MgO were first prepared and investigated. As an inert oxide additive, magnesium oxide with a specific surface area of 324 m^2^/g and a grains size of 5.1 nm was used. Due to a high specific surface area and a strong surface basicity, the organic salt is likely to have a strong adhesion to the MgO surface and spreads along its surface just at the first heating of the initial salt–oxide mixture. As a result of the strong adhesion, the salt transforms into an interface-stabilized amorphous state within the thin layer near the interface. The analysis of the DSC data allowed one to estimate the concentration and the thickness of this amorphous layer as 4.8 nm. The amorphous interface phase has an enhanced ionic conductivity. As a result, the conductivity of the nanocomposite increases with the concentration of the amorphous phase and reaches 1.1 × 10^−3^ S/cm at 150 °C at a concentration of the MgO additive x = 0.90 corresponding to the maximum content of the amorphous phase. The conductivity of the nanocomposite is by three orders of magnitude higher than the conductivity of pure Bu_4_NBF_4_. The nanocomposites are electrochemically stable below the voltage of nearly 2.5 V that allows them to use solid electrolytes in solid-state supercapacitors working at low voltages. A relatively low decomposition voltage of the electrolyte is likely to be caused by the presence of OH groups on the MgO surface or BF_4−x_OH_x_^−^ anions in the electrolyte. At high concentrations of MgO when the total volume of the salt is low the composites become nano- and mesoporous. The information obtained in the present work may be useful in the synthesis and applications of other hybrid nanocomposite systems.

## Figures and Tables

**Table 1:** Atomic concentration of elements and atomic ratios determined from XPS spectra in pure components and the 0.08Bu_4_NBF_4_-0.92MgO composite.

Sample	Elements, Atomic Percent						Atomic Ratio			
	Mg	B	C	N	O	F	F/B	N/C	O/Mg	Mg/C
MgO	32.6	0.0	34.3	0.0	33.1	0.0	0.0	0.000	1.0	0.95
Bu_4_NBF_4_	0.0	4.9	66.4	4.2	2.3	22.2	4.5	0.063	-	-
0.08Bu_4_NBF_4_-0.92MgO	24.1	2.4	40.0	0.0	24.2	9.3	3.9	0.000	1.0	0.60

**Table 2:** Values of experimental conductivity parameters *E*_a_ and log(*A*) for pure salt Bu_4_NBF_4_ and (1-x)Bu_4_NBF_4_–xMgO composites.

x	0	0.3	0.5	0.7	0.85	0.9	0.95
*E*_a_, eV	1.00 ± 0.01	0.89 ± 0.02	0.98 ± 0.02	1.01 ± 0.02	0.93 ± 0.02	0.92 ± 0.02	0.95 ± 0.02
log(*A*, S·cm·K^−1^)	8.32 ± 0.10	7.99 ± 0.10	9.40 ± 0.10	10.3 ± 0.2	10.3 ± 0.2	10.6 ± 0.2	10.0 ± 0.2

## Data Availability

The detailed results and experimental samples may be delivered by on request.
